# Bilocular Stomach

**Published:** 1913-02

**Authors:** 


BILOCULAR STOMACH.—G. Jeanneney, *Jour. de Med. de Bordeaux,* September 20, 1912, refers to case of his own previously reported, occuring in a woman of 74 and due to a corset. He now presents a specimen from Dupoux's practice, occuring in a woman aged 54, who died of cerebral haemorrhage. He does not specify the cause of the present case but sums up the general etiology as follows: Corset pressure, frequent in acquired cases; Sappey's theory of energetic muscular contraction of the stomach, untenable; reversion to ancestral type, in congenital cases, among which the present may belong. Testut stated that rodents generally had bilocular stomachs. The author concedes this for the rabbit, although the division is not conspicuous externally. In dissections of the young rat, he had not found this condition. In ruminants, the stomach of the foetus is bilocular, the quadrilocular type being developed later.

